# Temozolomide alleviates breast carcinoma via the inhibition of EGFR/ERK/ MMP-1 pathway with induction of apoptotic events

**DOI:** 10.1590/acb391624

**Published:** 2024-05-24

**Authors:** Weijun Zhu, Fengjun Zhang, Maoyun Wang, Shuai Meng, Fang Ren

**Affiliations:** 1 Taizhou Municipal Hospital – Department of Pathology – Zhejiang Province, Taizhou Zhejiang, China.; 2 The 940th Hospital of Joint Logistics Support Force of PLA – Department of Mammary Gland – Lanzhou, Gansu, China.; 3 First Medical Center of PLA General Hospital – Department of Traditional Chinese Medicine – Beijing, China.

**Keywords:** Temozolomide, Apoptosis, Breast Neoplasms, Drug Therapy

## Abstract

**Purpose:**

To evaluate the chemotherapeutic activity of temozolomide counter to mammary carcinoma.

**Methods:**

In-vitro anticancer activity has been conducted on MCF7 cells, and mammary carcinoma has been induced in Wistar rats by introduction of 7, 12-Dimethylbenz(a)anthracene (DMBA), which was sustained for 24 weeks. Histopathology, immunohistochemistry, cell proliferation study and apoptosis assay via TUNEL method was conducted to evaluate an antineoplastic activity of temozolomide in rat breast tissue.

**Results:**

IC_50_ value of temozolomide in MCF7 cell has been obtained as 103 μM, which demonstrated an initiation of apoptosis. The temozolomide treatment facilitated cell cycle arrest in G2/M and S phase dose dependently. The treatment with temozolomide suggested decrease of the hyperplastic abrasions and renovation of the typical histological features of mammary tissue. Moreover, temozolomide therapy caused the downregulation of epidermal growth factor receptor, extracellular signal-regulated kinase, and metalloproteinase-1 expression and upstream of p53 and caspase-3 proliferation to indicate an initiation of apoptotic events.

**Conclusions:**

The occurrence of mammary carcinoma has been significantly decreased by activation of apoptotic pathway and abrogation of cellular propagation that allowable for developing a suitable mechanistic pathway of temozolomide in order to facilitate chemotherapeutic approach.

## Introduction

Mammary carcinoma is the most recurrent carcinogenic disorder and significantly causes death for women globally, as per the World Health Organization. According to the GLOBOCAN 2020, mammary carcinoma is the most often diagnosed malignancy, with an appraised 2,261,419 recently stated cases and 684,996 died in 2020^1^. The leading carcinoma type in women is breast cancer, which totalizes 18% of the global breast cancer incidences^2^. The modification of cellular physiology and transduction ways of normal cell led to the carcinogenic manifestation through the abnormal mutation of several regulatory proteins, thus causing cellular propagation.

Members of the epidermal growth factor receptor (EGFR) family were discovered as some of the most important cancer molecular targets to date^3^. Breast cancer patients who have EGFR overexpression have larger tumors, poorer clinical outcomes, and poorer differentiation^4^. Although EGFR overexpression is seen in all breast cancer subtypes, the particularly aggressive triple-negative breast cancer and inflammatory breast cancer exhibit EGFR overexpression more commonly^5^.

Epithelial-mesenchymal transition (EMT) is hypothesized to facilitate cancer cell migration and invasion in a number of malignancies when EGFR mutations are present^6^. The Ras-ERK (extracellular signal-regulated kinase) pathway has been demonstrated to control EMT, tumor invasion, and metastasis downstream of EGFR. It is known that, when ERK activates RSK, cancer cells exhibit mesenchymal motility and invasion^7^. Furthermore, transforming growth factor-beta signaling has been linked to ERK; it has been demonstrated that transforming growth factor-beta1 promoted EMT by activating ERK1^8^.

Breast cancer incidence or invasiveness was found to be correlated with matrix metalloproteinase-1 (MMP-1) in several cancer types^9^^,^ 10. In terms of physiology, this enzyme is essential for normal tissue re-modeling processes such mammary gland involution, ovulation, angiogenesis, and wound healing^11^. Additionally, several diseases, including cancer invasiveness, have been associated to excessive expression of MMPs. The pathophysiologic significance of MMP-1 in the advancement of metastatic disease is being further investigated considering evidence from numerous clinical investigations. Moreover, increased MMP-1 expression in tissues with atypical ductal hyperplasia may act as a signal for identifying people who will develop invasive breast cancer^12^. In addition, several studies reported the EGFR has a vital part to control the MMP-1 activity through Ras/MEK/ERK pathway in malignant cell invasion and progression^13^^,^ 14.

Temozolomide (TMZ) is an alkylating drug of the second-generation member of the imidazotetrazine and exhibits activity against a variety of human carcinoma cell lines and xenografts^15^. In the treatment of glioblastoma, it serves as the first-line anti-cancer medicine. It is typically administered together with radiation, then concurrently with or after TMZ^16^. Furthermore, Wang et al. reported that the treatment with TMZ significantly causes the downstream of ERK signaling pathway on proliferation and cellular motility of glioblastoma C6 cells. From this study, it has been suggested that TMZ therapy in combination with ERK1/2 inhibitors could be more emergent therapeutic strategy for the management of glioblastoma^17^.

According to certain evidence, the dosage of TMZ affects how the host immune system responds. For instance, in mice and humans, transitory lymphodepletion brought on by the injection of higher dosages of TMZ boosted an immunotherapy-induced specific anticancer immune response while also boosting the number of regulatory T cells (Tregs)^18^.

The efficacy of standard and metronomic dosages of TMZ in association with anti-programmed cell death protein-1 (anti-PD-1) treatment on modifying the incidence of peripheral immune-repressive cells, such as Myeloid-derived suppressor cells (MDSCs) and Tregs, was also discussed by Karachi et al.^19^. Interestingly, the conventional dose of TMZ administration caused the induction of programmed death-ligand 1 (PD-L1) mutation on the surface of cancer cells in association with elevated immunosuppressive activity^20^. In addition, TMZ has been found to be significantly prevented the brain metastases of murine breast cancer model in combination with Trastuzumab emtansine (T-DM1)^21^.

A phase II clinical trial for TMZ has been conducted (NCT00005054) in women with advanced mammary carcinoma to determine the anticancer activity by preventing the tumor cell growth. Another randomized phase II clinical trial (NCT05128734) of TMZ alone or along with olaparib in patients with suffering triple negative mammary carcinoma (TNBC) is under progress. Moreover, TMZ in combination with T-DM1 has also been evaluated (NCT03190967) to determine if the combination therapy lowers the risk of brain metastases in HER2-positive mammary carcinoma patients. A phase II clinical investigation determined the effectiveness and safety of irinotecan as combined by TMZ in breast cancer patients. The results demonstrated that the combination of irinotecan and TMZ has been well tolerated, exhibited some clinical action across various mammary carcinoma subtypes along with developing central nervous system disorders and offers a reasonable opportunity for patients who does not participate for further radiation or clinical research^22^. The experimental evidence also suggested that TMZ therapy increased the oncolytic virotherapy in both human and murine breast cancer cells^23^. On the other hand, another phase II clinical trial of TMZ on metastatic breast cancer executed thru National Cancer Institute of Canada-Clinical Trials Group observed that there was no effect of TMZ on the metastatic breast cancer including brain metastases on heavily pretreated women^24^.

Despite the recent advancements, the activity of TMZ has been limited due to its toxicity, poor solubility, and hydrolyzation. Moreover, the blood–brain barrier and molecular and cellular heterogeneity and therapy resistance have limited the therapeutic effects of TMZ in treating glioblastoma, which mandates TMZ encapsulation in nanocarriers to increase TMZ stability, half-life, biodistribution, and efficacy^25^.

The current research is the first one that investigated the role of the growth factor regulation for the treatment of mammary cancer by TMZ through both in-vitro and in-vivo strategies. Till now, there is absence of documented confirmation that indicates anticancer activity of TMZ via the initiation of apoptosis and regulation of growth factors on mammary carcinoma model. Thus, the current investigation has aimed on the TMZ directed chemotherapeutic assessment on mammary carcinoma via in-vivo and in-vitro experimentation.

## Methods

### Chemicals and reagents

TMZ (#T2577), 3,3ʹ- diaminobenzidine (DAB) (#D12384), biotinylated goat antirabbit IgG (#21537), proteinase K (#1.07393), and streptavidin peroxidase (#S5512) have been purchased through Sigma Aldrich Chemical Co. (St. Louis, MO, United States of America). EGFR (#GTX121919), ERK (#GTX635617), MMP-1 (#GTX100534), proliferating cell nuclear antigen (PCNA) (#GTX100539), and Anti-mouse p53 (#GTX70214) were procured through GeneTex International Corporation (Global). Apoptotic assay kit (#MK500) has been purchased by Takara Bio Inc. (Kusatsu, Japan). Additional supplementary chemicals have been obtained through the local suppliers at its pure form.

### In-vitro assessment

#### Cell culture

MCF7 (HTB-22) mammary carcinoma cells were purchased from American Type Culture Collection (ATCC), United States of America, which were supplemented with Dulbecco’s modified eagle medium (DMEM) growth media, 10% fetal bovine serum (FBS), 100-μg/mL streptomycin, and 100 U/mL penicillin. The cultured cells have been further incubated in 5% CO_2_ and 95% relative humidity at 37°C.

#### Cellular viability assessment

The cellular viability analysis has been carried out through MTT (3-(4,5 dimethylthiozol-2-yl)-2,5-diphenyl tetrazolium bromide) assay. The MCF7 cell line has been seeded into 96-microtiter well plate and incubated in 5% CO_2_ at 37°C for 24 h. After that, the cell line was introduced in various concentrations (10–200 μM) of TMZ, which further incubated for 24 h. Subsequently, 0.5 mg/mL of MTT solution in DMEM was introduced into the cells and incubated for 3 h. Then, the MTT solution with dimethylsulphoxide (DMSO) was supplemented due to dissolve the formazan crystal, and absorbance was measured at 560 nm. The percentage of cellular viability was calculated through Eq. 1:


%cellularviability=[Meanabsorbanceoftemozolomidetreatedcells/Meanabsorbanceoftemozolomideuntreatedcells]×100 (1)

The IC_50_ value was estimated through linear regression Eq. 2:


y=mx+C (2)

Where: y = 50.

The value of m and C was estimated through the cellular viability graph.

#### Flow cytometric assay

The cancerous cell apoptosis was evaluated through flow cytometric assay with Annexin V-FITC apoptotic cells detection kit. The MCF7 cell line was seeded into six well plates and introduced in various concentrations including IC_25_, IC_50_ and IC_75_ of TMZ for 24 h. Later, the cell line was labelled by Annexin V-FITC and propidium iodide (PI). Then, the mammary cells was assessed via FACSCelesta flow cytometer (BD Biosciences, San Jose, CA, United States of America), and the cellular cycle phases regulation was also demonstrated. The outcomes of the study have been analyzed thru Modfit tools to estimate the percentage of apoptotic cells.

#### Determination of caspase-3 proliferation

The caspase-3 proliferation on MCF7 cells was evaluated via flow cytometric analysis. The cells was seeded into six well plates and introduced in various concentrations including IC_25_, IC_50_, and IC_75_ of TMZ for 24 h. After that, the anti-rabbit caspase-3 polyclonal antibody was introduced into cells for 30 min in unlighted state at 37°C. The % of cells labelled by the caspase-3 antibody was analyzed through flow cytometry assay.

#### Western blot

The EGFR, ERK, and MMP-1 expression on the MCF-7 cell line has been determined by Western blotting analysis. The cancerous cell was incubated in various concentrations (IC_25_, IC_50_, and IC_75_) of compound for 24 h. The cancerous cell lysate obtained was fixed in SDS-PAGE (8-12%) electrophoresis, which was transferred to polyvinylidene difluoride (PVDF) membrane. The PVDF membrane was incubated overnight thru anti-rabbit EGFR, ERK, and MMP-1 antibodies (1:500 dilution). After that, this membrane was incubated by goat anti-rabbit secondary antibody that was HRP-conjugated, and chemiluminescent kit was used to analyze the expression, in which β-actin served as the loading control.

### In-vivo assessment

#### Animals

Experimental animal was conducted as per the Institutional Animal Ethical Committee of First Medical Center of PLA General Hospital (approval no. 2023TMZ251). The entire techniques accomplished during the study was complied by the Animal Research Reporting of In-Vivo Experimental guidelines, which was encompassed in institutional ethical rules. The female Wistar rats (70–120 g) of 6-week-old were found by First Medical Center of PLA General Hospital Animal Center for the chemotherapy study. The entire experimental animals were kept in polypropylene cages, along with free supply of adequate food and drinking water with 50–58% relative humidity at 22 ± 3°C and 12 h light/dark sequences. The experimental animals were acclimatized for one week prior to the starting of the experiment.

#### Experimentation protocol

Followed by the acclimatization, animals was taken arbitrarily into five groups, and each group consisted of six rats. Wistar rat female, 7- to 8-week-old (excluding group 1) was introduced 7, 12-dimethylbenz(a)anthracene (DMBA) (0.5 mg / 100 g body weight) in corn oil via tail vein single intravenous injection, and the mammary carcinoma was persuaded. Subsequently the administration of chemical carcinogen DMBA, the treatment with TMZ in experimental animals was done via oral gavage and continued for six months. All experimental animal groups were alienated as followed by:

- Group 1: normal (vehicle) control group;
- Group 2: induction of DMBA, which was represented as carcinogen control group;
- Group 3: DMBA persuaded breast carcinoma + administered with 50 mg/kg of TMZ;
- Group 4: DMBA introduced breast carcinoma + treated with 100 mg/kg of TMZ;
- Group 5: DMBA persuaded breast carcinoma + treated with 200 mg/kg of TMZ.

At the termination of chemotherapeutic analysis, the experimental animals were euthanized, and mammary tissues were isolated and conserved into 10% neutral buffered formalin (NBF) solution for fixation. Mammary tissues were evaluated to demonstrate the cancer architecture. Breast tissues were assessed through histological, immunohistochemical, and cellular proliferation analysis.

### Histological assessment of the mammary tissue

The experimental animals were euthanized. Then, the initial cut was completed at the ventral midline from the inguinal nipples to thoracic nipples along with skin and without incised the peritoneum, thus the breast tissues were isolated. The mammary tissues were fixed in 10% NBF solution for 24 h. After that, the isolated tissues were dehydrated and embedded with paraffin and scotched into 5-micron-thickness glass slides. Then, the mammary tissue sections were stained with hematoxylin and eosin (H&E) and observed under light microscope for histopathological examination.

### Antioxidant assessment of the mammary tissue

Mammary tissues were homogenized (10% w/v) into 0.1 M of phosphate buffer saline at pH 7 for the evaluation of antioxidant activity. After that, these homogenized mammary tissues were centrifugated for 15 min, and the supernatant was separated for the enzymatic assessment^26^.

### Catalase assessment

In accordance with Sinha et al., the homogenized mammary tissues were further used for the evaluation of catalase (CAT) activity. Absorbance was observed at 620 nm, and the CAT activity was quantified by μmol of H_2_O_2_ consumed/min/mg protein^27^.

### Superoxide dismutase assessment

The superoxide dismutase (SOD) activity of homogenized mammary tissues was studied as the protocol revealed by Awasthi et al. SOD action was anticipated by units/min/mg protein^28^.

### Glutathione peroxidase assessment

The glutathione peroxidase (GPx) action was estimated in accordance with the protocol devised by Rotruck et al. GPx action was described by μmol of GSH consumed/min/mg of protein^29^.

### Immunohistochemical assessment

The excised breast tissues were embedded into paraffin wax and scorched into 5-micron thickness, which was fixed onto the poly-L-lysine embedded glass slide. Followed by the mammary tissues deparaffinization, the glass slides were engrossed into H_2_O_2_ solution. Then, the breast tissue sections were treated by protein blocking solution for 1 h. After that, the sections were incubated with anti-rabbit EGFR (1:500 ratio), ERK (1:500 ratio), MMP-1 (1:500 ratio), anti-mouse p53 (1:1,000), and anti-rabbit PCNA (1:500 ratio) antibodies for 24 h at 4°C. Then, the tissue sections were cleaned by phosphate buffer saline (PBS) and incubated with HRP conjugated secondary antibody for 30 min. DAB was applied on the tissue section for the color development, and counterstained was completed through the tissue sections stained with H&E.

Finally, the tissue sections were detected through light microscopic (Olympus CX 21i TR) analysis, as well as pictures were taken for assessment of immune responsive cells through ImageJ software (version 1.8.0). The red brown coloring of the cytoplasm/nucleus was considered positive staining (any other coloring was considered negative staining). Negative controls were used to avoid bias. For the negative controls, the primary antibody was omitted in each different immunohistochemistry staining.

### Apoptotic analysis through TUNEL assay

The mammary tissues were mounted on poly-L-lysine embedded glass slide and treated with proteinase K (20 μg/mL) in PBS at 22–25°C for 10 min, as a resulting to digest the non-specific proteins. The peroxides quenching was carried out through the tissue section treated through H_2_O_2_ (3% in PBS) at 22–25°C for 5 min. After that, the sections were treated by terminal deoxynucleotidyl transferase (TdT) buffer solution (30 mM of Trizma base, pH 7.2, 1 mM of cobalt chloride, 140 mM sodium cacodylate, which was further treated with TdT solution including TdT and dUTP for 90 min at 37°C. The reaction of the tissue sections was obstructed by the introduction of 2% standard saline citrate for 10 min at 22–25°C. Then, the tissues were introduced in anti-digoxigenin peroxidase for 30 min at 22–25°C. DAB was applied on the tissue section for the color development, and counterstained was completed by H&E stain. Afterwards, the tissue section was observed through light microscopy for the analysis of apoptotic cells.

### Determination of apoptotic index and labeling index

The percentage of PCNA positive cells / total number of cells was denoted through labeling index (L1), and the percentage of TUNEL positive cells / total number of cells was represented by apoptotic index (AI).

### Statistical assessment

The experimental consequences were quantified through mean ± standard error mean. The one-way analysis of variance as post-hoc test (Tukey’s test) was performed to estimate the statistical importance through Graph pad prism software (Version 5). The differences was hypothecated to be statistically significant with p < 0.05.

## Results

### In-vitro assessment

#### Cell viability assay

**Figure 1:**
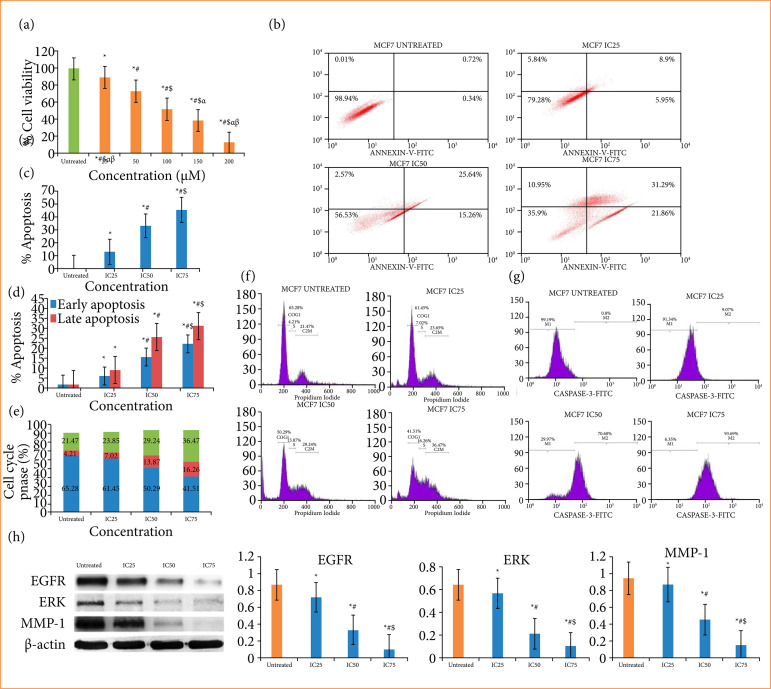
Effect of temozolomide on MCF7 cell line at 24 h. **(a)** Activity of temozolomide on cell viability where * denotes p < 0.05 as comparison with untreated group, as well as

Cell viability analysis of TMZ demonstrated a significant inhibition of cell viability dose dependently (Fig. 1a). The cell viability of MCF7 cells was found to be 88.56, 73.28, 48.53, 37.98, and 10.25% at the concentration of 25, 50, 100, 150 and 200 μM, respectively. Then, the IC_50_ value of TMZ was quantified to be 103 μM. The succeeding doses for the chemotherapeutic research were further quantified as 51.5 (IC_25_), 103 (IC_50_), and 154.5 (IC_75_).

#### Flow cytometry

The treatment with TMZ showed a higher % of apoptotic cells in MCF7 cell line dose dependently (Figs. 1b and 1c). The % apoptotic cells were determined as 15.64, 38.24, and 52.81% at IC_25_, IC_50_ and IC_75_ of the compound. The cellular population was also superior in dose-dependent manner of early and late apoptotic stage followed by the treatment with TMZ (Fig. 1d).

The outcomes of the flow cytometry study for cell cycle phases distribution are shown in Figs. 1e and 1f. A significant increase in the cellular population at G0/G1 phase, S phase and G2/M phase was observed in TMZ therapy group. The cell population at Go/G1 phase was quantified as 65.28, 61.45, 50.29, and 41.51% in untreated group, IC_25_, IC_50_ and IC_75_ concentrations, correspondingly. Likewise, the cellular propagation at S phase and G2/M phase was estimated as 4.21, 7.02, 13.87, 16.26 and as 21.47, 23.85, 29.24, and 36.47% in untreated group, IC_25_, IC_50_ and IC_75_group, correspondingly.

#### Temozolomide enhanced caspase-3 expression

The management by TMZ suggested a dose dependently increment of the caspase-3 mutation in MCF7 cells (Fig. 1g). The untreated group demonstrated the caspase-3 marked cells at M1 quadrant, while a significantly superior population of caspase-3 marked cells at M2 quadrant were denoted in the cells introduced in TMZ.

### Consequence of temozolomide on EGFR, ERK, and MMP-1 expression

The study of Western blot represented the TMZ treated group substantially abrogated the EGFR, ERK, and MMP-1 expression in MCF-7 cell as comparison to untreated cells (Fig. 1h).

### Histopathological assessment of mammary tissue

**Figure 2:**
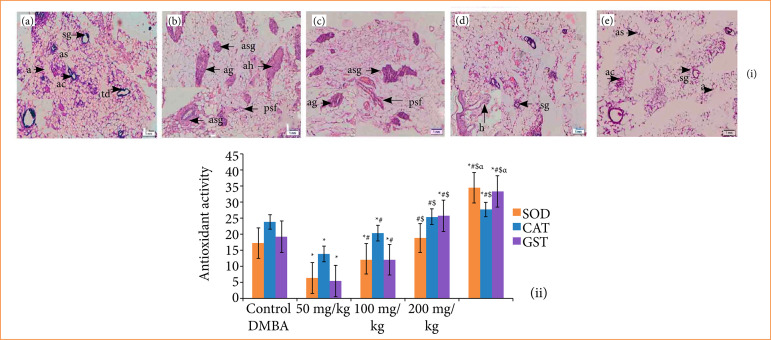
**(i)** Histopathological assessment of breast tissue of **(a)** normal control group displaying the alveoli **(a)**, alveolar septa (sg), terminal duct lobular units (td), serous gland (sg), Acinus (ac). **(b)** DMBA induced group displays atrophy of glands through periductal stromal fibrosis and fatty tissue (psf), atrophy of serous glands (asg) with adjacent stromal fibrosis, atrophy of glands (ag) with adjacent fatty tissue, atypical hyperplasia (ah). **(c)** Fifty-mg/kg temozolomide treated group in DMBA induced breast cancer displaying atrophy of glands (ag), atrophy of serous glands (asg), and periductal stromal fibrosis and fatty tissue (psf). **(d)** One hundred-mg/kg temozolomide treated group in DMBA induced breast cancer displaying hyperplasia of mucinous and serous glands **(h)**. **(e)** Two hundred-mg/kg temozolomide treated group in DMBA induced breast cancer showing almost normal morphology. **(ii)** Temozolomide activity on in-vivo antioxidant enzymes like SOD, GST, and CAT.

SOD: superoxide dismutase; CAT: catalase; GST: glutathione-S-transferase; DMBA: 7, 12-dimethylbenz(a)anthracene; *significant variance as compared with normal control group (p < 0.05); #significant variance as comparison with carcinogen control group (p < 0.05); $significant difference as comparison with 50 mg/kg group (p < 0.05); αsignificant variance as an associated with 100 mg/kg group (p < 0.05). Source: Elaborated by the authors.

Histopathological assessment of mammary tissue was depicted in Fig. 2i and Table 1. The vehicle control group (group 1) showed the typical morphological characteristics of breast tissue that displayed the alveoli (*a*), acinus (*ac*), terminal duct lobular units (*td*), serous gland (*sg*), and alveolar septa (sg) of breast tissues (Fig. 2ia). The DMBA control group (group 2) demonstrated the atrophy of glands through the periductal stromal fibrosis and fatty tissues (*psf*), atrophy of serous glands (*asg*) bounded by stromal fibrosis, atrophy of glands (*ag*) bounded by fatty tissues, and atypical hyperplasia (*ah*) (Fig. 2ib). Fifty-mg/kg dose of TMZ administered group represented the atrophy of glands (*ag*), atrophy of serous glands (*asg*), and periductal stromal fibrosis and fatty tissues (*psf*) (Fig. 2ic). One hundred-mg/kg dose of TMZ treated group showed the hyperplasic lesions of mucinous and serous glands (*h*) (Fig. 2id). Nevertheless, the 200-mg/kg treated group elicited an astonishingly downstream of hyperplasia, as well as cell proliferation of mammary tissue (Fig. 2ie), which showcased the normal morphological architecture of breast tissue.

**Table 1:** Semi-quantitative histology scoring of breast tissue*.

	A	B	C	D	E
Periductal stromal fibrosis	0	3	3	1	0
Atrophy of serous glands	0	3	2	2	0
Atrophy of glands	0	3	3	1	0
Atypical hyperplasia	0	3	3	0	0

* Histological appearance of lung tissue;

A: vehicle control, B: carcinogen control; C: 50 mg/kg temozolomide; D: 100 mg/kg temozolomide;E: 200 mg/kg temozolomide. Semi-quantitative histology scoring system: 0: normal; 1: slightly abnormal; 2: moderately abnormal; 3: maximally abnormal. Source: Elaborated by the authors.

### Antioxidant status

Carcinogen control group exhibited the drastically decreases of glutathione-S-transferase (GST), CAT, and SOD intensities via comparison with vehicle control group (p < 0.05). TMZ treated group showed a higher intensity of GST, CAT, and SOD, considerably in mammary tissue, wherein 200-mg/kg treated group displayed substantial upstream effect of antioxidants in mammary tissue as comparison with vehicle control and other TMZ treated groups (p < 0.05) (Fig. 2ii).

### Immunohistochemical assessment

**Figure 3:**
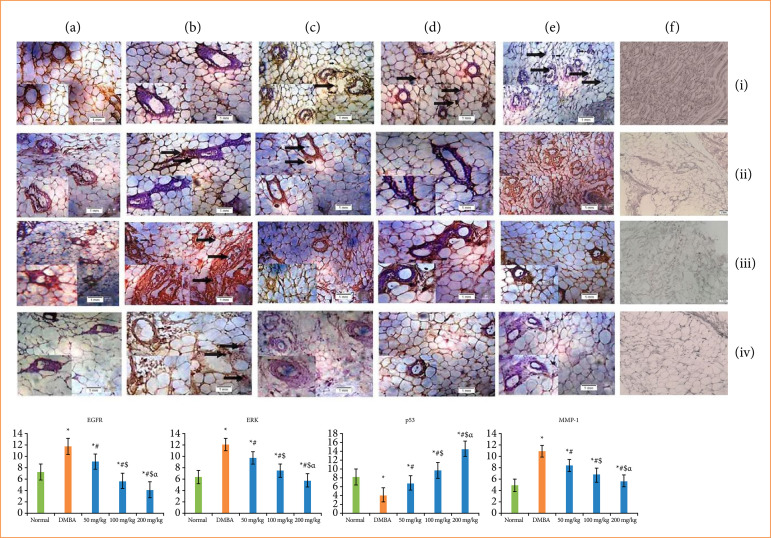
Immunohistochemical assessment of **(i)** p53, **(ii)** EGFR, **(iii)** ERK and **(vi)** MMP-1 expressions in mammary tissues of several groups of rats, including **(a)** normal control, **(b)** carcinogen control, **(c)** 50 mg/kg of temozolomide treated, (d and e) 100 and 200 mg/kg temozolomide treated, **(f)** negative control. All images were showed at 40X.

EGFR: epidermal growth factor receptor; ERK: extracellular signal-regulated kinase; MMP-1: metalloproteinase-1. Source: Elaborated by the authors.

Immunohistochemical assessment of mammary tissue was accomplished for the evaluation of protein manifestation that comprises p53 (cancer repressive protein), EGFR, ERK, and MMP-1 (Fig. 3). Vehicle control group (group 1) showed a higher proliferation of p53 in epithelial tissues of alveolar ducts and terminal end bud (Fig. 3ia), while DMBA control group (group 2) showed a lower mutation of p53 (Fig. 3ib). TMZ administered with different doses such as 50-, 100-, and 200-mg/kg body weight a remarkably *(p < 0.05)* increases p53 proliferation was noticed at terminal end bud, as well as alveolar zone (Figs. 3ic–3ie) as comparison with DMBA control group.

The moderate proliferation of EGFR was identified in cap cells of vehicle control group and terminal end bud region (Fig. 3iia). In DMBA control group, the EGFR mutation was dramatically increased (Fig. 3iib). However, the treatment of TMZ at various doses (50, 100, and 200 mg/kg) were depicted a sustainable (p < 0.05) inferior the EGFR mutation in terminal end buds, as well as alveolar duct area (Figs. 3iic–3iie).

The normal control group showed an incredible ERK proliferation in breast tissue (Fig. 3iiia) at terminal end buds, while a lower proliferation was represented at the cap cell layer. However, ERK propagation was considerably higher in DMBA persuaded carcinogen control group (Fig. 3iiib) as the hyperplastic lesions of breast tissues since comparison with vehicle control group.

On the other hand, a substantial (p < 0.05) decreases of the ERK mutation was detected in TMZ administered group (Figs. 3iiic–3iiie) (50, 100, and 200 mg/kg). Besides that, MMP-1 proliferation was noted in intact terminal ducts region, as well as the cap cells of vehicle control group (Fig. 3iva). The expression of MMP-1 was drastically amplified in carcinogen control group (Fig. 3ivb), while TMZ administered groups (50, 100, and 200 mg/kg) demonstrated a remarkably (p < 0.05) decrease of the MMP-1 proliferation in terminal end buds and alveolar duct zone (Figs. 3ivc–3ive). However, the negative control was depicted in Figs. 3if–ivf.

### Cellular proliferation analysis

**Figure 4:**
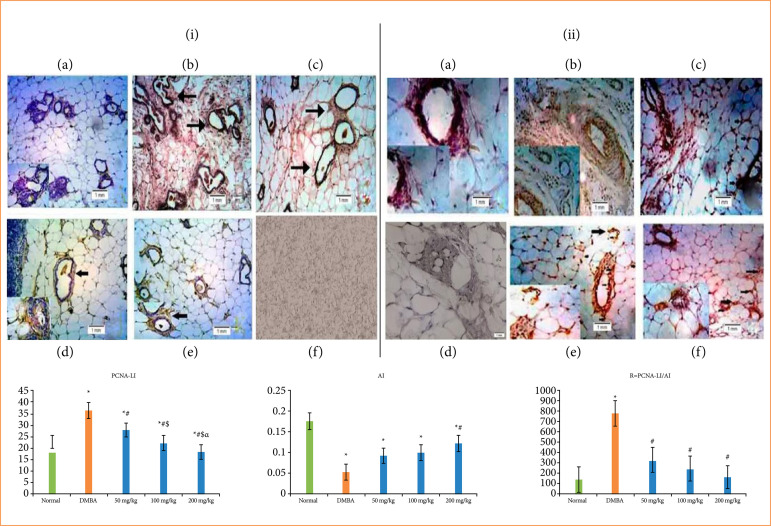
**(i)** The immunohistochemical assessment of alteration of PCNA of several group of rats: **(a)** normal control, **(b)** carcinogen control, **(c)** 50 mg/kg temozolomide treated, (d and e) 100 and 200 mg/kg temozolomide treated, **(f)** negative control. All images were visualized at 40X. **(ii)** Apoptotic assessment by TUNEL method: **(a)** normal control, **(b)** carcinogen control, **(c)** 50 mg/kg temozolomide treated, (d and e) 100 and 200 mg/kg temozolomide treated, **(f)** negative control. All images were showed at 40X.

PCNA: proliferating cell nuclear antigen; LI: labelled index; DMBA: 7, 12-Dimethylbenz(a)anthracene; AI: apoptotic index. Source: Elaborated by the authors.

The action of TMZ administration in cell propagation of mammary tissue were depicted in Fig. 4i, which represents the chemotherapeutic activity of TMZ in DMBA persuaded breast carcinoma. A distinctive nuclear localization and brown color formation caused by chromogen introduction were presented in the PCNA labeling cells, that been associated with the quantification of cells. In vehicle control group, they designated the lower signs with PCNA marked cells (Fig. 4ia). The percentage of the PCNA marked cell was quantified through labeling index (LI), in which carcinogen control group (Fig. 4ib) expressed a higher LI. Conversely, a drastically (p < 0.05) decrease of the LI was denoted in TMZ administered group (Figs. 4ic–4ie) (50, 100, and 200 mg/kg). The negative control is illustrated in Fig. 4if.

### Apoptotic assay through TUNEL method

TUNEL analysis was carried out for demonstrating the manifestation of apoptotic events in cells with hyperplastic abrasions of breast tissue. The apoptosis events in cells were recognized through the brown coloration caused by the chromogen introduction (Fig. 4ii). DMBA persuaded carcinogen control group (Fig. 4iib) denoted the decrease amount of TUNEL responsive cells, typically three to five apoptotic cells, in an around 700 cells, as compared with vehicle control group (Fig. 4iia). An extensively (p < 0.05) higher mutation of TUNEL responsive apoptotic cells was showed in TMZ treatment group (Figs. 4iic–4iie) (50, 100, and 200 mg/kg) in associated with DMBA control group. Usually eight or nine apoptotic cells, in an around 700 cells, were noticed in TMZ treatment group. Herein, the overlapping happens between the condensed chromatin of apoptotic cells and brown stain that associated with the TUNEL assessment through the initiation of apoptotic events. The negative control is illustrated in Fig. 4iif.

The % of TUNEL responsive cells was determined via the apoptotic index (AI). The significance of R was designated by the ratio of cellular propagation to apoptosis. The elevated R value in carcinogen control group was represented by the higher proliferative action of cancerous cells, but the TMZ treated group exerts significantly reduced R value caused by the initiation of apoptosis.

## Discussion

Recently, the antiquated chemotherapeutic drugs have become outdated owing to their various adverse activities, as well as the development of drug resistance that assigning the advancement of new drugs to contract malignant prevalence even though evading the allied side events. Furthermore, the natural products may be the utmost sustainable possibility as of their prospective antineoplastic effects and less adverse events^30^. Therefore, the chemotherapeutic activity of TMZ on DMBA persuaded breast cancer in Wistar rat model has been examined, and probable molecular mechanistic pathway of temozolomide as an antineoplastic agent has been further hypothesized.

The chemotherapeutic activity of TMZ was investigated against MCF7 breast carcinoma cell line. Previously, the TMZ activity on the cellular viability of MCF7 and SKBR3 cell line has been evaluated, and TMZ reduced the cell viability by 50% of both the cell line at 40 and 50 μM concentration, respectively^31^. In this study, the cell viability assessment demonstrated the compound significantly prevented the viability of MCF7 cells dose dependently. Moreover, the IC_50_ value was also considered through the cellular viability analysis that was obtained as 103 μM. In flow cytometry assay, it represented an induction of apoptosis on MCF7 cells by the cell line treated with TMZ.

The significant number of apoptotic cells was observed in TMZ introduced group, in which the 154.5-μM compound treated group demonstrated a substantially higher percentage of apoptosis (52.81%) in MCF7 cell line. The dysregulation of the normal cell cycle regulation since the abnormal cell proliferation is one of the vital characteristics of the malignant cells^32^. Hence, the cell cycle phases distribution with an initiation of cell cycle arrest is a foremost approach for the chemotherapeutic study, which facilitates for the accomplishment of the DNA damage and provokes apoptotic pathway in cancerous cells. The TMZ activity on cell cycle phases distribution was evaluated on MCF7 cells by the flow cytometry assay. The outcome of the cell cycle study demonstrated as the cellular arrest at S and G2/M phases of cell cycle which eventually activated by the apoptotic induction in breast carcinoma cells with TMZ treatment.

The initiation of carcinoma via chemical carcinogens is a multiphase method that is concerned with activating of normal cells to cancer cell and then allowing those cells to invade into surrounding tissue^33^. In this investigation, the breast carcinoma has been manifested in Wistar rat model by the management of DMBA, that was resembled with human mammary carcinoma. Together with the histology, hyperplastic evolution in the pre-carcinoma and carcinoma lesions is moderately similar with the human mammary carcinoma prevalence^34^. DMBA administered in experimental rats caused the formation of hyperplastic abrasions, thus changed the normal architectural association with breast tissue due to the higher amplification of cellular propagation, which was further evaluated by histological assessment. TMZ treatment fruitfully reversed the mammary tissue’s cellular architectural modifies to normalcy by preventing the initiation of hyperplastic abrasions, which reduced the cell propagation that noticeably demonstrates the chemotherapeutic effect of drug on mammary carcinoma.

The anticancer activity of TMZ was evaluated by the establishment of experimental animal model counter to breast cancer and is greatly valued to investigate the human breast carcinoma as rat breast has extraordinary propensity for developing cancer that is similar with human breast carcinoma^35^. The recent research depicts antineoplastic activity of TMZ on DMBA persuaded mammary carcinoma via the interruption of cancer cell dissemination, that triggers the apoptosis and decreases the mammary carcinoma occurrence through downstream effect of EGFR, ERK, and MMP-1 expression and the activation of apoptotic indicators p53 in rat mammary carcinoma model. Additionally, this study delivers a summary of antineoplastic mechanism of this new chemotherapeutic drug.

The immunohistochemical assessment of mammary tissue was conducted to examine the alteration of p53 and survival and cellular proliferation proteins like EGFR, ERK, and MMP-1 to inaugurate the signaling pathway by which TMZ demonstrates its anticancer efficacy to counteract the breast cancer. Numerous revisions stated the alteration of tumor suppressor protein (p53), which is directly connected with cancer cell owing to the modification of cancer suppressive ways^36^. During the stimulation of the intrinsic apoptosis ways that leads to start the caspase-dependent downregulation of cancer proliferation and ultimately results in cell death, increased p53 expression reduced the number of cells^37^^,^ 38.

The outcomes of this investigation prompted the overexpression of p53 in the TMZ treated group that supports to initiation of apoptosis in mammary cancer cells. Besides that, the carcinogen control group showed a substantially lower p53 expression, which disrupts the apoptosis pathway in the malignant cells and allows the cancer cells to grow deliberately. Additionally, the EGFR/ERK transduction scheme promotes cellular growth and inhibits malignant cell death via a number of cellular survival pathways that are facilitated with MMP-1.

In the current research, the carcinogen control group displayed EGFR, ERK, and MMP-1 overexpression, which leads to the uncontrolled cell proliferation of the cancer cells due to the induction of cell survival pathways, whereas TMZ treated group displayed a substantial decreased in EGFR, ERK, and MMP-1 proteins expression in mammary carcinoma cells. As a result of TMZ treatment, carcinoma cells involve significant decreases of cell propagation and become more susceptible to p53 mediated apoptosis.

The reactive oxygen species (ROS) shows an essential part in progression of cellular oxygen metabolism, which interprets several cellular effects via stimulations of many signaling ways that accountable for cell progression and explosion at physiological concentrations. Nevertheless, ROS amplification causes the distraction of redox homeostasis that leads to ROS mediated damage of some essential macromolecules such as proteins, DNA and lipids^39^. Thus, it eventually promotes the carcinogenesis^40^.

Normal cells are enabling to defend themselves from the undesired activity of oxidants through the effects of several antioxidant enzymes like CAT, SOD and GST^41^. It has been observed that both normal and cancer cells depend on the activation of the nuclear factor erythroid 2-related 2 (Nrf2) transcriptional networks to neutralize the oxidative insult by controlling the redox homeostasis via upregulating the expression of antioxidant defense genes^42^.

The TMZ treatment had shown to increase Nrf2 expression in cancer cells which negatively regulates ROS generation by promoting Nrf2 mediated expression of GSH^43^^,^ 44. Moreover, TMZ treatment also showed a high increase in Sp1 levels and upregulated SOD levels, which eventually lowered intracellular ROS levels^45^. In the present study, it has been established that the DMBA control group displayed the declined activity of enzymes like CAT, SOD, and GST, whereas TMZ treated group displayed an upstream of antioxidant signs in mammary tissue that concern with the anticipation of ROS generation along with hinders the cell expression and dissemination.

Uncontrolled cellular proliferation is a crucial indicator of cancer occurrence. The modulation of various mechanistic ways that operate the cell cycle machinery is necessary for the control of cellular propagation. The cellular propagation is regulated by the several checkpoint pathways. In this research, the TMZ treated group showed a substantial reduce in cellular proliferation, which represented its anticancer effect on mammary carcinoma model. The cellular proliferative action of malignant cells in mammary tissue was evaluated through PCNA label. The entire % of PCNA marked cells was considered through LI, in which the distinctive nuclear localizations were observed in cells marked through PCNA. As a result of DMBA induced carcinogen control group, a superior LI was noticed wherein subsequently lowered the LI through TMZ administration since observed in TMZ treated group.

Due to the early phase of mammary carcinoma, apoptotic event plays a crucial part in the prevention of tumorigenesis. In this investigation, TUNEL analysis was implemented for the evaluation of apoptosis initiation in carcinoma cells caused by the TMZ treatment. The management of cancer with TMZ (50, 100, and 200 mg/kg) treatment demonstrated a higher % of cells entering into apoptotic events in which a substantial lower apoptotic index was seen in DMBA persuaded carcinogen control group.

**Figure 5:**
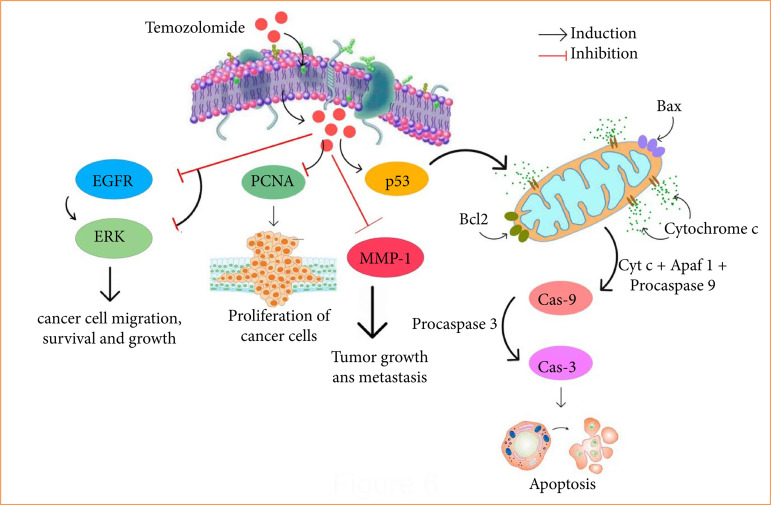
The molecular mechanistic pathway of temozolomide in mammary carcinoma.

EGFR: epidermal growth factor receptor; ERK: extracellular signal-regulated kinase; MMP-1: metalloproteinase-1; PCNA: proliferating cell nuclear antigen. Source: Elaborated by the authors.

Considering all the results of this investigation, the chemotherapeutical action of TMZ on DMBA persuaded rat breast cancer with the prevention of cancer cell expression and propagation along with the initiation of apoptosis through the upstream expression of p53 and downstream expression of EGFR, ERK, and MMP-1 remains intensely recognized (Fig. 5).

Additionally, this research extremely affords new insight of prospective chemotherapy at a drug therapy regimen with a noticeably low dose in order to inhibit the expansion of cancer via reorganization of possible biomarkers that could be linked to apoptosis in breast carcinoma through activating intrinsic apoptotic signaling path.

Despite these remarkable findings, there are certain limitations in this research work that need to be addressed, such as absence of power calculation and explanation of the sample size. In addition, the pharmacophore analysis must be carried out to identify more chemotherapeutic targets, and more target proteins should be evaluated to establish a firm mechanistic pathway of cancer progression and to observe the intracellular alteration upon drug treatment. The number of cell lines evaluated for chemotherapeutic study should be also increased.

## Conclusion

The newer chemotherapeutic strategies in contest with mammary cancer are instantly directive as in the last years a little improvement was attained in this field, even though the accessibility of novel, as well as superior chemotherapeutic interventions.

In the present research, the chemotherapeutic activity of TMZ on DMBA persuaded the breast cancer due to the modulation of EGFR/ERK/MMP-1 signaling way in rat model was exhibited. Subsequently, as an appropriate evaluation of the limits of this recent research, antineoplastic activity of TMZ should be confirmed through the preclinical study, thus additionally assessed for potential drugs substitution in clinical research in nearby future prospective.

## Data Availability

All the dataset were generated or analyzed in the current study.
